# Defining a murine ovarian cancer model for the evaluation of conditionally-replicative adenovirus (CRAd) virotherapy agents

**DOI:** 10.1186/s13048-019-0493-5

**Published:** 2019-02-15

**Authors:** Rebeca González-Pastor, Ahmad Mohammad Ashshi, Adel Galal El-Shemi, Igor P. Dmitriev, Elena A. Kashentseva, Zhi Hong Lu, S. Peter Goedegebuure, Osvaldo L. Podhajcer, David T. Curiel

**Affiliations:** 1 The Division of Cancer Biology and Biologic Therapeutics Center, Department of Radiation Oncology, School of Medicine, Washington University in Saint Louis, 660 South Euclid Avenue, Campus Box 8224, St. Louis, MO 63110 USA; 2 Department of Laboratory Medicine, Faculty of Applied Medical Sciences, Umm Al-Qura University, PO Box 7607, Holy Makkah, Saudi Arabia; 3 Department of Pharmacology, Faculty of Medicine, Assiut University, Assiut, Egypt; 4 Department of Surgery, Washington University School of Medicine, Saint Louis, MO 63110 USA; 5 Alvin J. Siteman Cancer Center, 660 S. Euclid Avenue, St. Louis, MO 63110 USA; 6 Laboratory of Molecular and Cellular Therapy, Instituto Leloir, IIBBA-CONICET, Buenos Aires, Argentina

**Keywords:** Adenovirus, Virotherapy, Ovarian cancer, CRAd, ID8, Anti-tumor immunization

## Abstract

**Background:**

Virotherapy represents a promising approach for ovarian cancer. In this regard, conditionally replicative adenovirus (CRAd) has been translated to the context of human clinical trials. Advanced design of CRAds has sought to exploit their capacity to induce anti-tumor immunization by configuring immunoregulatory molecule within the CRAd genome. Unfortunately, employed murine xenograft models do not allow full analysis of the immunologic activity linked to CRAd replication.

**Results:**

We developed CRAds based on the Ad5/3-Delta24 design encoding cytokines. Whereas the encoded cytokines did not impact adversely CRAd-induced oncolysis in vitro, no gain in anti-tumor activity was noted in immune-incompetent murine models with human ovarian cancer xenografts. On this basis, we explored the potential utility of the murine syngeneic immunocompetent ID8 ovarian cancer model. Of note, the ID8 murine ovarian cancer cell lines exhibited CRAd-mediated cytolysis. The use of this model now enables the rational design of oncolytic agents to achieve anti-tumor immunotherapy.

**Conclusions:**

Limits of widely employed murine xenograft models of ovarian cancer limit their utility for design and study of armed CRAd virotherapy agents. The ID8 model exhibited CRAd-induced oncolysis. This feature predicate its potential utility for the study of CRAd-based virotherapy agents.

## Background

Virotherapy is a targeted therapy which has been applied to carcinoma of the ovary [1, 2]. In this strategy, a virus is engineered to replicate selectively in tumor target cells while sparing normal tissues. This selectively of replication thereby induces targeted tumor cell killing, a process termed “oncolysis”. To this end, a range of viruses have been engineered as virotherapy agents including Herpes Simplex virus, polio virus, measles virus, adenovirus, and others [3–6]. Of note, a disproportionate number of virotherapy interventions in humans have been applied for the context of cancer of the ovary, reflecting pharmacologic advantages which accrue the possibility of intraperitoneal delivery.

We have pursued a strategy of virotherapy for ovarian cancer exploiting adenovirus [7–9]. In this regard, the unique molecular plasticity of adenovirus has allowed us to modify native viral tropism to accomplish target cell infection via over-expressed tumor markers [10, 11]. Specifically, we have developed a conditionally replicative adenoviral agent (CRAd) with the integrin-binding peptide RGD4C incorporated at the capsid fiber binding protein [12]. This modified virus can thus infect targets via integrins recognized by the capsid incorporated RGD4C binding motifs. This is especially relevant as ovarian cancer tumor cells express a paucity of the native adenovirus receptor coxsackie-and-adenovirus receptor (CAR) [13, 14]. The capacity to accomplish “CAR-independent” infection circumvents tumor cell resistance to adenovirus infection thereby dramatically improving the oncolytic potency of our advanced generation CRAd agents [15]. Of note, we have carried-out a Phase I human clinical trial with our infectivity enhanced CRAd, Delta-24-RGD, and demonstrated its safety as well as its virologic efficacy [16].

More recently, it has been shown that virotherapy agents may achieve anti-tumor activities over-and-above their direct oncolytic effects. Specifically, it appears that virus-mediated oncolysis may represent an effective means to induce anti-tumor immunization [17–19]. A number of recent studies have sought to define the biologic basis of this vaccine effect [20, 21]. In addition, strategies to exploit this biology have been incorporated into virotherapy agent design. For adenovirus-based virotherapy agents, this has involved “arming” the CRAd with an immunomodulatory molecule (ex. GM-CSF, IL-12) in an effort to optimize immunologic milieu to enhance CRAd-based anti-tumor immunization [22–24].

Despite the novel opportunities offered by this approach, full development is currently limited by the lack of models that allow full characterization of CRAd-based anti-tumor immunization. In this regard, studies of CRAd interventions for cancer of the ovary have been endeavored almost exclusively in orthotopic human tumor xenograft models [25]. This is owing to the fact that cross-species factors have been understood to limit productive infection by human adenovirus of murine target cells. On this basis, it has heretofore not been feasible to exploit syngeneic immunocompetent murine cancer models to study CRAd-based anti-tumor immunization.

Very recently, however, it has been recognized that the biologic block to human adenovirus replication in murine cells is not absolute; human adenovirus can achieve some level of binding, entry, and post-entry replication in selected murine targets [25, 26]. On this basis, investigative groups have re-visited the utility of syngeneic immunocompetent murine models to gain insight into the anti-tumor immunizing potentials of adenoviral virotherapies [27–29]. Most recently, Jiang et al. have found that the murine glioma cell line GL261 could support limited CRAd-based replication and oncolysis. On this basis they were able to exploit an immunocompetent murine model of glioma and gain key insights into CRAd-based anti-tumor immunization [30]. Other recent reports have also exploited murine cell lines moderately permissive for human adenovirus replication to employ in corresponding immunocompetent murine models of cancer for studies of CRAd-induced anti-tumor immunization [31, 32]. These new understandings of the utilities of immunocompetent murine models now potentially provide a rational pathway to design CRAd agents fully exploiting tumor immunobiology.

On the basis of these considerations, we have sought to advance an ovarian cancer CRAd design strategy based upon immunobiologic arming methods. Despite the fact that we could design and rescue CRAds with immunobiologic arming molecules, little augmentation in anti-tumor activity was noted in standard orthotopic xenograft models of ovarian cancer. On this basis we have sought to establish the possibility of exploiting available syngeneic immunocompetent murine models of cancer of the ovary. In this report we demonstrate that the widely available ID8 model of ovarian cancer supports adenovirus-mediated cytolysis, a key attribute allowing its potential employ for study of CRAd agents engineered to exploit tumor immunobiology. This syngeneic immunocompetent model now can be explored to allow rational design of CRAd agents for the achievement of optimal anti-cancer immunotherapy via oncolytic virotherapy.

## Methods

### Cells

The human ovarian carcinoma cell line SKOV3.ip1 was obtained from Janet Price (M. D. Anderson Cancer Center, Houston, Tex.). The mouse epithelial ovarian cancer cell line ID8, which was originally established by Dr. Kathy Roby (Kansas University Medical Center) and its derivative expressing firefly luciferase were obtained from Dr. Robin Bachelder (Duke University Medical Center) and were maintained in DMEM (high glucose, Gibco-Life Technologies) containing 4% FBS, 1× penicillin and streptomycin as described elsewhere [33]. The ID8-*Trp53*^*−/−*^ [34] mouse ovarian surface epithelial cells (F3) were kindly provided by Dr. Iain McNeish (University of Glasglow) and labeled to express mCherryLuciferase (F3mCherryLuc). Both cell lines were grown in DMEM (high glucose, Gibco-Life Technologies) containing 4% FBS, 1× penicillin and streptomycin and 1× ITS (Insulin-Transferrin-Selenium solution) (Gibco-Life Technologies). The 911 human embryonic retinoblasts derived by transformation with a plasmid containing 79–5789 bp of the Ad5 genome [35] were obtained through Crucell Holland B.V. (Leiden, The Netherlands). The human lung carcinoma cell line A549 were obtained from American Cell Type Culture Collection (ATCC, Manassas, Virginia USA). All cell lines were grown at 37 °C in medium recommended by the suppliers in a humidified atmosphere of 5% CO_2_.

### CRAd vectors

The replication-competent CRAd Delta 24 was provided by Dr. J. Fueyo. (The University of Texas M. D. Anderson Cancer Center, Houston, TX). This virus contains a 24-nucleotide deletion, from Ad5 bp 923 to 946 (both included), corresponding to the amino acid sequence L_122_TCHEAGF_129_ of the E1A protein known to be necessary for Rb protein binding [36]. Details of the tumor-specific replication of this virus are presented elsewhere [37, 38]. The incorporation of the RGD-4C motif, known to interact with α_v_ integrins, into the HI loop of the fiber knob (T_546_CDCRGDCFCP_547_) to enhance Delta24 CRAd infection efficiency of tumor cells was described previously [15, 39]. The construction of Ad5/3-Delta24 CRAd, which contains the fiber knob domain replaced with its counterpart from Ad serotype 3 (Ad3) was described elsewhere [9, 40]. The construction of Ad5/3-Delta24-based CRAd-IL24 and CRAd-ING4 vectors expressing human IL-24 [41] or ING4 (the inhibitor of growth 4) [42] gene, respectively, under transcriptional control of human cytomegalovirus (CMV) immediate-early promoter/enhancer incorporated in place of the deleted E3B region was described in detail recently [43]. Non-armed control CRAd that encodes the secreted Gaussia luciferase (Gluc) from the copepod *Gaussia princeps* (New England BioLabs Inc., Ipswich, MA USA) driven by CMV promoter in place of E3B region was described recently [43]. Wild-type Ad5 was kindly provided by Dr. H Ugai (Washington University in St Louis, St Louis, MO).The replication incompetent Ad5∆E1 containing the CMV promoter-driven firefly luciferase reporter gene in place of the deleted E1A/B genes was described before [44] and propagated using 911 cells. All CRAd vectors and wild type Ad5 were propagated using A549 cells, purified by centrifugation on CsCl gradients according to standard protocol, and dialyzed against phosphate-buffered saline (PBS) (8 mM Na_2_HPO_4_, 2 mM KH_2_PO_4_ [pH 7.4], 137 mM NaCl, 2.7 mM KCl] containing 10% glycerol. The titers of physical viral particles (vp) were determined by the methods of Maizel et al. [45]. The titers of infectious viral particles were determined by plaque assay using 911 cells as described by Mittereder et al. [46].

### Analysis of virus-mediated cytotoxicity in vitro

To monitor cytotoxic effects induced by Ad5, Delta-24, or Delta-24-RGD vector the cell monolayers grown in 96-well plates (3 × 10^3^ to 5 × 10^3^ cells/well) were infected in triplicates with each virus at MOI of 100 vp/cell. The infected and mock-infected cells were subjected to CellTox™ Green Cytotoxicity assay as recommended by the manufacturer (Promega Corporation, Madison, WI) by adding DNA-binding cyanine dye on day 1 and monitoring the increase in fluorescent signal intensity, which is proportional to cell lysis, till day 5–6 post-infection. The degree of virus-mediated cell killing was measured using the Synergy-HT plate reader (Bio-Tek Instruments, Winooski, VT) equipped with 485 nm excitation and 520 nm emission wavelength filters and the average values of relative fluorescent units (RFU) are shown. The relative cell viability on day 5–6 post-infection with either Ad5, Delta-24, Delta-24-RGD or non-replicating Ad5∆E1 control was determined using the Cell Proliferation Assay (Promega Corporation, Madison, WI) as recommended by the manufacturer. Assay was performed by adding 10 μL CellTiter 96 AQ_ueous_ One Solution Reagent directly to culture wells containing red phenol free media supplemented with 2% FBS, incubating for 2 h and then recording the absorbance at 490 nm with a plate reader (Synergy HT, Bio-Tek Instruments, Winooski, VT). The data are presented as the percentages of viable cells in monolayers infected with each viral dose that were determined with respect to the uninfected control set as 100%.

### S.C. Tumor xenograft model

Female 5–7 week-old athymic nu/nu mice (The Jackson Laboratory) were kept under pathogen-free conditions according to the American Association for Accreditation of Laboratory Animal Care guidelines. Animal protocols were reviewed and approved by the Institutional Animal Care and Use Committee of the Washington University in Saint Louis, School of Medicine. Five million SKOV3.ip1 cells were xenografted s.c. into the right flank of the mice under anesthesia. When the nodules reached a volume of 60–70 mm^3^, a single Ad dose (10^10^ vp in 20 μl of PBS) or the same volume of PBS was administered intratumorally (*n* = 9–10 animals/group) and injections were repeated weekly for 3 consecutive weeks. Tumor size was monitored twice a week, and fractional volume was calculated by standard technique for volume determination of subcutaneously xenografted tumors in vivo using external caliper measurements and the modified ellipsoid formula 1/2 (Length × Width^2^) [47, 48]. Animal were euthanized according to IACUC policy “Maintaining Tumors in Rodents Policy” if the tumor became greater than 2 cm in any dimension or showed clinically significant cutaneous ulceration.

### I.P. Syngeneic orthotopic model

Female 5–7 week-old C57Bl/6 mice (The Jackson Laboratories) were injected i.p. with 5 × 10^6^ F3mCherryLuc cells in 200 μL PBS. After one week, mice were injected i.p. with 1 × 10^10^ vp of wild-type Ad or replication-incompetent Ad vectors in 100 μL PBS (*n* = 9). Mice were euthanized after 17 days of tumor inoculation. In vivo bioluminescence imaging was performed on days 7 and 17 after tumor inoculation on an IVIS Lumina (PerkinElmer, Waltham, MA; Living Image 3.2, 1 s exposure(s), 8 bin. Mice were injected intraperitoneally with D-luciferin (150 mg/kg in PBS; Gold Biotechnology, St. Louis, MO) and imaged using isoflurane anesthesia (2% vaporized in O_2_). Total photon flux (photons/sec) was measured from fixed regions of interest (ROIs) over the entire mouse abdomen using Living Image 2.6. Animal protocols were reviewed and approved by the Institutional Animal Care and Use Committee of the Washington University in Saint Louis, School of Medicine.

### Statistical analysis

All data are presented as the mean ± SD. The Student’s two-tailed *t*-test was used to determine statistical significance at the 95% confidence level, with *p* ≤ 0.05 being considered significantly different for in vitro data. The animal survival data from the tumor xenograft model were analyzed using product-limit method to estimate the survival function S(*t*) for each treatment and control group with IBM-SPSS statistics software. The long rank test with family-wise significance level *α* = 0.05 was employed to compare animal survival between control group treated with non-armed CRAd and each armed CRAd treatment group. Mann-Whitney test was used to determine statistical significance in the syngeneic orthotopic model groups with non-parametric distribution, with *p* ≤ 0.05 being considered significantly different for the syngeneic orthotopic model.

## Results

### Anti-tumor effects of armed CRAd agents in murine xenograft model of carcinoma of the ovary

We have advanced the development of advanced generation CRAd agents which embody the capacity to accomplish CAR-independent infection of tumor cell targets for enhanced oncolytic potency. Infectivity enhancement was initially embodied into CRAd design via incorporation of the integrin-binding peptide RGD-4C in the HI loop of the fiber knob [49, 50]. We next pursued a strategy of chimerism for the fiber knob by replacing the CAR-binding adenovirus serotype 3 fiber knob with the corresponding fiber knob domain of the human adenovirus serotype 3 [9, 40]. Our studies confirmed that this latter strategy also allowed significant enhancement of oncolytic potency in murine orthotopic xenograft models of ovarian cancer virotherapy. On the basis of these findings, we sought to advance the design of our knob 3 infectivity-enhanced CRAd via an “arming” strategy. Specifically, we sought to configure within the adenovirus genome the immunoregulatory molecules ING and IL-24.

**Fig. 1:**
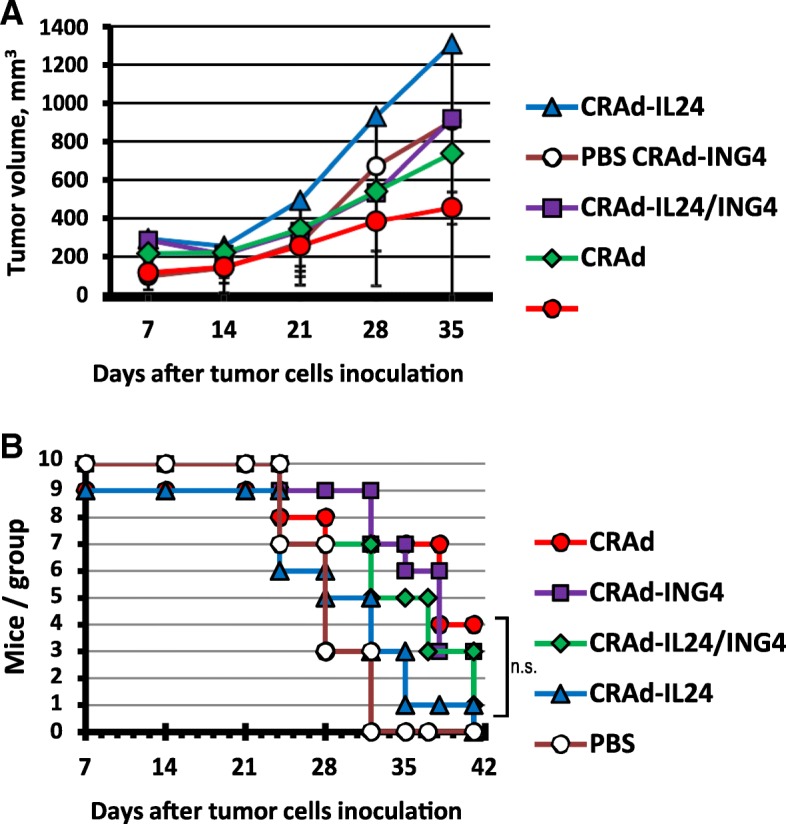
Analysis of tumor growth inhibition following armed CRAd administration. Subcutaneous tumor xenografts were established on rear flank of female nude mice using SKOV3.ip1 cells. Tumor nodules were directly injected with 10^10^ vp (5 × 10^8^ pfu/dose) of either CRAd-IL24, CRAd-ING4, CRAd-IL24/ING4 (5 × 10^9^ vp of each armed CRAd), control CRAd or PBS alone on day 7 and injections were repeated weekly for 3 consecutive weeks. **a** Mean tumor volume of each group is shown through day 35 after tumor implantation. Each data point represents the cumulative mean of tumor volumes (mm^3^) in each group while vertical error bars depict standard deviations. **b** Kaplan-Meier curves of overall survival of animals treated with the indicated armed CRAd vectors, non-armed CRAd control, or PBS are shown. Data analysis provides no evidence that survival experience observed in mice treated with either CRAd-IL24, CRAd-ING4, or CRAd-IL24/ING4 (the indicated armed CRAd vectors) is significantly different from non-armed CRAd control (n.s.)

On this basis we constructed Ad5/3-Delta24 CRAds armed with either ING4 or IL-24 [43]. These earlier studies confirmed that the armed CRAds expressed the encoded arming genes at a high level. Importantly, modification of the Ad5/3-Delta24 CRAds in this manner was not deleterious to their anti-tumor oncolytic properties. We thus evaluated these armed CRAds in an established murine orthotopic xenograft model of human cancer of the ovary which employed the human ovarian cancer cell line SKOV3.ip1. Analysis of treated animals was endeavored for tumor growth and survival. As can be seen, the armed versions of Ad5/3-Delta24 CRAd exhibited less anti-tumor activity than the parental agent. Importantly, this observation was noted with both assays of tumor growth (Fig. 1a) and survival (Fig. 1b).

### Cytotoxicity induced by replication-competent human adenovirus

The findings noted in our study of the armed Ad5/3-Delta24 CRAds in the murine orthotopic xenograft model highlighted the limits of immunodeficient models for analysis of the immunobiologic activities of adenovirus-based virotherapy agents. To address this limit, we considered the potential utility of the murine immunocompetent syngeneic ID8 model of human carcinoma of the ovary. Of note, an expanding repertoire of reports have recently highlighted the utility of this model for study of ovarian cancer immunobiology. More importantly, this model has demonstrated utility for analysis of immunotherapy interventions for cancer of the ovary [51].

As noted, a number of recent reports have reported the utility of immunocompetent murine models to study the ability of CRAds to accomplish effective anti-tumor immunization [29, 30]. Whereas not fully permissive for productive human adenovirus replication, selected murine tumor cells targets appear to allow a level of human adenovirus replication of sufficient magnitude to activate anti-tumor immunity in the context of implantation in corresponding immunocompetent syngeneic murine models. On this basis, we studied the cytolysis in the murine cell lines ID8 and ID8luc, which were employed widely as a murine immunocompetent model of ovarian cancer [51].

**Fig. 2:**
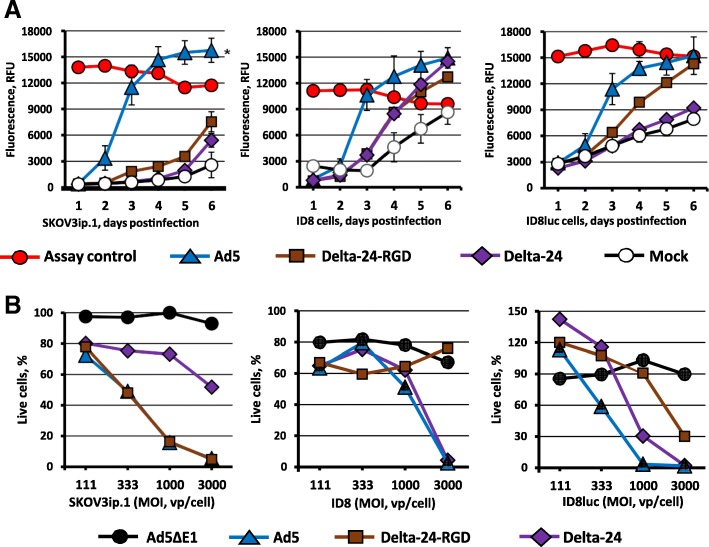
Analysis of cytopathic effects of replication competent Ad vectors. **a** Cytotoxic effects of wild type Ad5, Ad5-based Delta-24 and Delta-24-RGD CRAd vectors were compared in established ovarian cancer cell lines of human (SKOV3ip.1) and mouse (ID8 and ID8luc) origin using CellTox™ assayat the MOI of 100 vp/cell as compared to mock-infected cells (negative control) and completely lysed cells (positive assay control). Each data point represents the three replicate experiments ± SD (error bars are smaller than the symbols, *p ≤* 0.05 *). **b** Monolayers of SKOV3ip.1, ID8, and ID8luc were infected with indicated Ad vectors and cell viability determined on day 6 post-infection by adding CellTiter 96 AQ_ueous_ One Solution Reagent. The percentages of live cells in the monolayers exposed to replication-deficient E1-deleted Ad5∆E1 are shown as compared to wild type Ad5, Delta-24, and Delta-24-RGD-infected cells

We also employed in these studies the human ovarian cancer cell line SKOV3.ip1, with the wild type adenovirus Ad5 and the armed CRAd agents. These adenoviruses achieved prominent cytotoxic effects in this replication permissive tumor cancer cell line, as predicted (Fig. 2). The specificity of this effect was confirmed by the lack of toxicity induced by the replication-incompetent control adenovirus. Of note, similar effects were noted with analysis of the murine ovarian cancer cell lines ID8 and ID8luc. Indeed, the levels of induced tumor cell cytotoxicity were nearly comparable in the murine targets as noted in the human tumor targets.

**Fig. 3:**
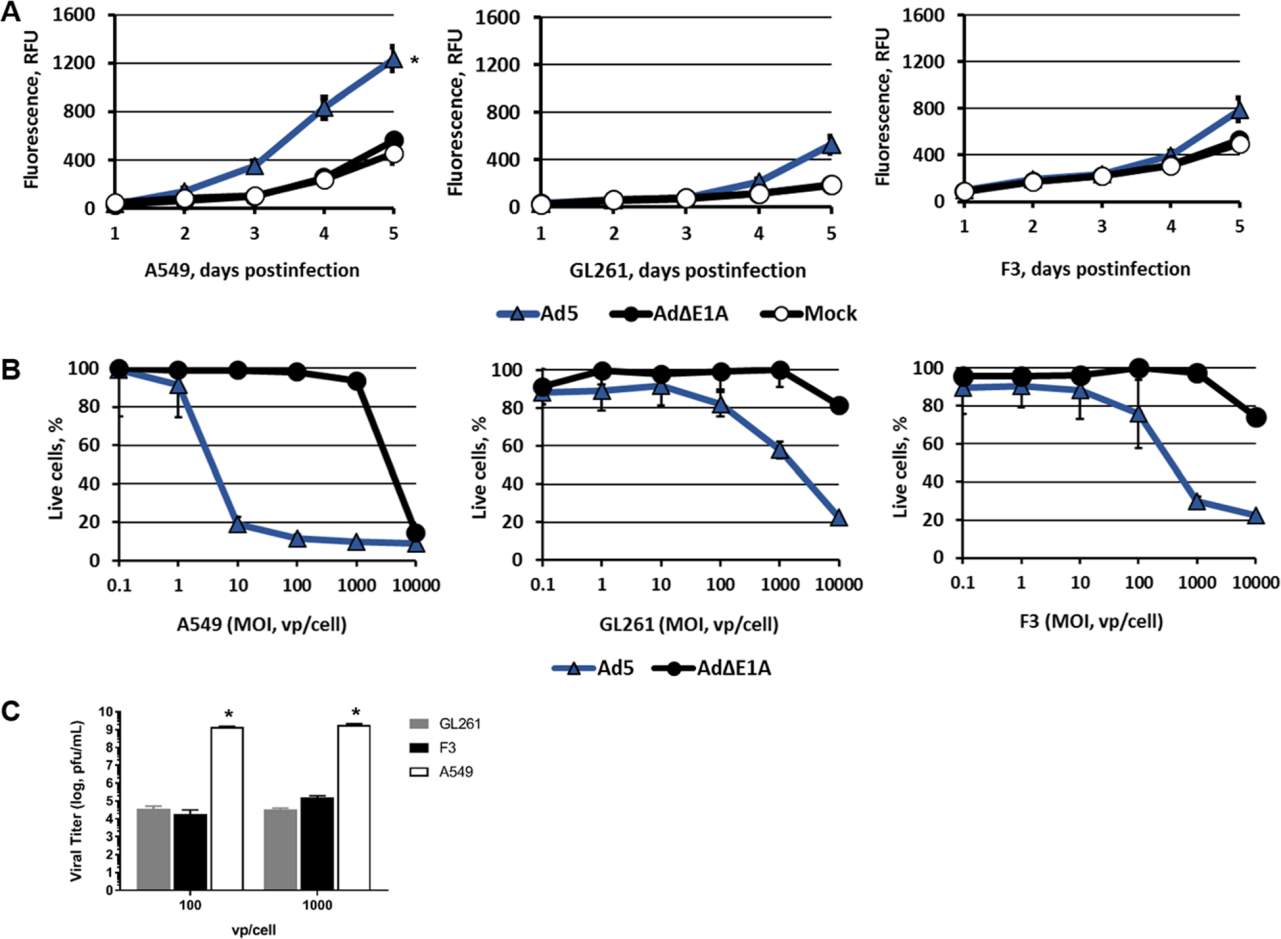
Analysis of cytopathic effects of replication competent Ad vectors. **a** Cytotoxic effects of wild type Ad5 were compared in A549, GL261 and F3 using CellTox™ assay. Each data point represents the cumulative mean of triplicate measurements ± SD (*p ≤* 0.05 *). **b** Monolayers of A549, GL261 and F3 were infected with indicated Ad vectors and cell viability measured after 5 days by adding CellTiter 96 AQ_ueous_ One Solution Reagent. The percentages of live cells in the monolayers exposed to replication-deficient E1-deleted Ad5∆E1 are shown as compared to wild type Ad5-infected cells. **c** Replication was assed 48 h after infection at MOI 100vp/cell and 1000vp/cell using the Adeno-X-Rapid Titer kit. Final titers were determined at pfu/mL (*p ≤* 0.05 *)

Of note, it has recently been shown that knockout derivatives of ID8 exhibited significant alterations of the tumor microenvironment and reduced survival, with an overall closer resemblance with the human disease. Although the p53-knockout ID8 derivative cell line, F3, exhibited a lower virus-induced cytotoxicity and replication compared to a highly susceptible human cancer line, A549 (Fig. 3), the levels were comparable to those obtained with GL261, a mouse murine cell line that has shown utility as a syngeneic model vis-à-vis induction of anti-tumor immunization.

### Anti-tumor effects of ad in murine syngeneic model of carcinoma of the ovary

**Fig. 4:**
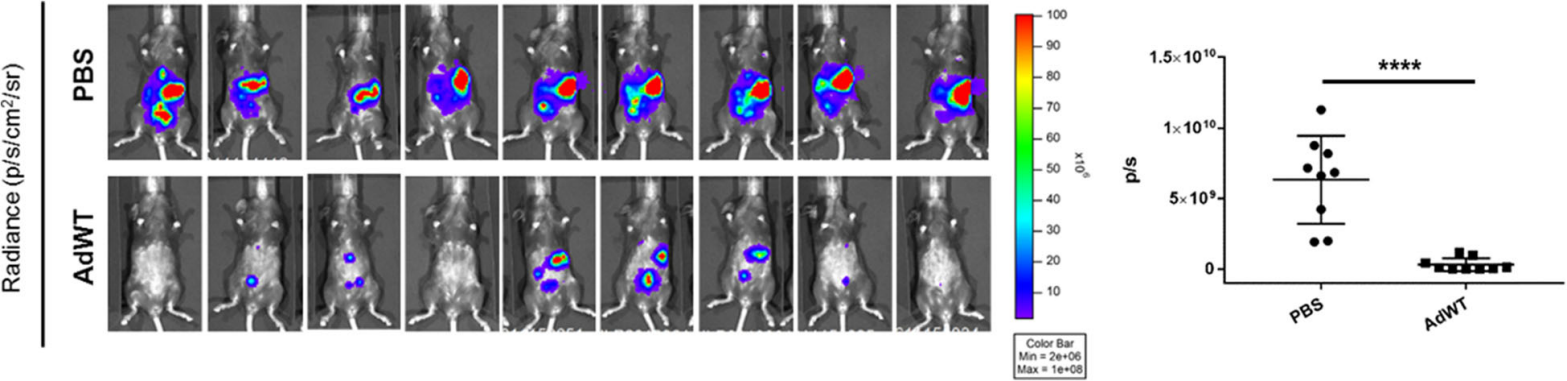
Analysis of tumor growth inhibition on a syngeneic orthotopic model following Ad administration. C57Bl/6 mice were injected i.p. with 5 × 10^6^ F3mCherryLuc cells. After one week, mice were injected i.p. with 1 × 10^10^ vp of wild-type Ad or replication-incompetent Ad vectorsBLI images and corresponding signal quantification 6 days after Ad administration. Significant differences were found between PBS and AdWT groups (*p* = 0.0001 ****)

Finally, we evaluated the of fully replication competent wild type adenovirus effect in a syngeneic murine model generated by the administration of F3mCherryLuc. Analysis of treated animals after 7 days of Ad5 injection showed a significant reduction on luminescence signal (Fig. 4). The ID8 line, and its derivative, thus exhibited: (1) a level of permissivity to adenovirus-mediated cytotoxicity in vitro comparable to GL261, a murine model useful for study of CRAd-induced anti-tumor immunization, and (2) susceptibility to adenovirus-mediated oncolysis in vivo. This initial study thus supports the employment of the F3 syngeneic murine model as a potentially robust system for the study of the therapeutic effect of CRAds for cancer of the ovary.

## Discussion

Whereas oncolytic virotherapy agents were originally designed to accomplish anti-neoplastic effects directly via viral replication and cytolysis, it has recently become apparent that they can also elicit potent multimodal immunogenic tumor cell death. In this regard, virus infection in cancer cells releases damage-associated patterns recognized by pattern recognition receptors expressed on cells of the innate immune system. Activation of these receptors induces pro-inflammatory cytokines provoking Th1-type immune responses [52–54]. Of note, such induced cytokines up-regulate MHC class I thereby increasing activity of this antigen presentation pathway [55]. The recognition of these potent immunostimulatory effects of virotherapy agents has led to design strategies to augment these vaccine effects [56]. Of note, our CRAd agent, Delta-24-RGD, has been adapted in this manner and shown to accomplish anti-tumor immunization in human clinical trials [16, 40, 57]. More recently, it has been noted that syngeneic immunocompetent murine cancer models can provide an effective system to ascertain vaccine gains which derive from CRAd agents [30]. This recognition now allows us to explore the murine ovarian cancer immunotherapy ID8 model to determine vaccine utilities that derive from our CRAds.

To this point, the species-specific restriction of human adenovirus has limited study of CRAds in available syngeneic immunocompetent murine cancer models. Specifically, the limited ability of human adenovirus to replicate in murine cells represented a practical impediment to study of the immunobiologic effects of CRAd agents. Efficacy in murine xenograft models, coupled to toxicology studies in C57BL6 mice, thus constituted the full translational rationale for human clinical trials. In these instances, however, the mandates of human trial design have not been compatible with fully testing hypotheses related to CRAd-based anti-tumor immunization.

A number of efforts have thus been advanced to circumvent this limit. In this regard, our group has adapted canine adenovirus type 2 (CAV-2) as a CRAd agent for study in canine concerns. We reasoned that such a “fully syngeneic” system would allow us to study CRAd biology in a genetically outbred and immunocompetent context with the highest level of analogy to the human context. Other groups have likewise pursued this approach and demonstrated its utility in guiding rational CRAd design [30]. This strategy, however, is limited to the availability of accrued canine patient cohorts and restricted to tumor types common in dogs. Another approach is based upon the employment of Syrian Golden Hamster (SGH). In this instance, the relative permissivity of SGH cells for human adenoviral replication provides an immunocompetent context to study replication-linked biologies relevant to CRAd design. As for the canine application, however, the limited cancer types available in the SGH model has limited wide application of this method.

Recently it has become apparent that the species restriction of human adenovirus is not absolute. In this regard, murine cells have been shown to support variable levels of binding, entry, and replication of human adenovirus. In selected indices, the full cycle of adenoviral replication can be achieved, albeit at lower levels than noted for fully permissible immortalized human cancer cell lines, such as HeLa and A549. Capitalizing on this recognition, groups have begun to apply human adenovirus-based CRAds in syngeneic immunocompetent murine cancer models. These reports clearly stablished that, in spite of the lower levels of adenovirus replication, clear anti-tumor immunization effects could be accomplished. Most importantly, these CRAd reports validated the link between CRAd replication and the agents’ vaccine effect. These observations now provide the possibility of exploiting such widely available murine cancer models to guide the design of CRAds for optimized anti-tumor immunization.

These considerations led us to consider the ID8 model of ovarian cancer to guide design and analysis of ovarian cancer CRAds. As noted, study of CRAds for cancer of the ovary has heretofore been endeavored exclusively in murine xenograft models. In this regard, the ID8 model represents a syngeneic immunocompetent model of carcinoma of the ovary with a human-like clinical presentation. These properties have led to its employ for a wide range of studies of tumor immunobiology and anti-cancer immunotherapies. In our current study we show ID8 supports human adenovirus mediated tumor cytolysis. This capability has been shown to be necessary and sufficient in a number of recent reports to allow study of CRAd-based anti-tumor immunization in other syngeneic immunocompetent murine cancer models. In addition, adenovirus-induced tumor cytolysis can accomplish a therapeutic effort in an orthotopic model context. We would note that the distinct delivery mandates of cancer of the ovary may present distinct barriers from those addressed in earlier studies of CRAds-based immunization in murine syngeneic models. Specifically, in these earlier studies CRAds were administered by direct intratumoral administration and with multiple dosing schemes. These protocols may have facilitated effective tumor cell cytolysis in vivo based in the high local levels of the CRAd agent achieved. For cancer of the ovary, the mandated intraperitoneal delivery schema may not allow comparable levels of tumor cell infection. These delivery barriers must be considered in the design of studies to ascertain anti-tumor immunization exploiting this model.

In the aggregate, the validation of these key feasibilities now rationalizes the use of this model to explore the full anti-tumor immunizing potential of the virotherapy approach for carcinoma of the ovary.

## Abbreviations

Ad: Adenovirus
Ad3: Adenovirus serotype 3
Ad5: Adenovirus serotype 5
BLI: Bioluminiscence imaging
CAR: Coxsackievirus group B and adenovirus receptor
CAV-2: Canine adenovirus type 2
CMV: Cytomegalovirus immediate-early promoter
CPE: Cytopathic effect
CR-2: Conserved region 2
CRAd: Conditionally replicative adenovirus
Gluc: *Gaussia princeps* luciferase
IL-12: Interleukin 12
IL-24: Interleukin 24
ING4: Inhibitor of growth 4 tumor suppressor protein
mAb: Monoclonal antibody
MDA-7: Melanoma differentiation associated gene 7
MOI: Multiplicity of infection
OvCa: Ovarian cancer
PBS: Phosphate-buffered saline
RGD-4C: Cys-Asp-Cys-Arg-Gly-Asp-Cys-Phe-Cys
SGH: Syrian golden hamster
vp: viral particles
